# Implementation of Primary Palliative Care in five Belgian regions: A qualitative study on early identification of palliative care needs by general practitioners 

**DOI:** 10.1080/13814788.2020.1825675

**Published:** 2020-10-20

**Authors:** Bert Leysen, Olivier Schmitz, Isabelle Aujoulat, Marlène Karam, Bart Van den Eynden, Johan Wens

**Affiliations:** a Primary and Interdisciplinary Care, University of Antwerp Faculty of Medicine and Health Sciences, Wilrijk, Belgium; b Institute of Health & Society (IRSS), Université catholique de Louvain Secteur des sciences de la santé, Bruxelles, Belgium; c Multidisciplinary Pain Centre, University Hospital Antwerp, Edegem, Belgium

**Keywords:** Palliative and terminal care, health care organisation and management, qualitative designs and methods, general practice/family medicine, general

## Abstract

Background

To deliver optimal palliative care, a Care Pathway for Primary Palliative Care (CPPPC) was developed. This CPPPC was implemented by general practitioners (GPs) in territories of five Belgian palliative care networks (2014–2016). Belgian doctors have much therapeutic freedom, and do not commonly follow guidelines.

Objectives

To assess how palliative care was provided by GPs before the CPPPC and its implementation project were presented publicly.

Methods

Between 2013 and 2015, seven focus groups with GPs were conducted. Participants included 15 GPs in three French-speaking focus groups and 26 GPs in four Dutch-speaking focus groups, with diversity for age, gender, palliative care experience and practice context. Some GPs implemented the CPPPC later.

Results

GPs considered each palliative care case unique and disliked strict protocols. However, they expressed a need for peer review and reflective frameworks. GPs felt it is important to identify palliative care patients ‘timely’, but found this difficult. Screening methods help, but are not widely used. GPs struggled most with identifying palliative care needs in non-oncological patients. Bad news breaking was considered difficult. Continuity of care was considered very important. However, advance care planning seemed more widely practised by Dutch-speaking GPs than by French-speaking GPs. The taboo of palliative care provoked emotional discussions.

Conclusion

Palliative care frameworks which help GPs to deliver ‘tailor-made’ care have more chance to be adopted than strict protocols. GPs should be given education for bad news breaking. Palliative care and advance care planning practices differ locally: guideline dissemination plans should respect these local differences.

## Introduction

KEY MESSAGES

- Tailor-made care is highly valued by GPs; this may complicate the application of (evidence-based) palliative care guidelines.
- Peer review discussions and practice-oriented education seem well-received educational strategies for quality improvement by GPs in palliative care.
- Engaging local health care providers is warranted in tailoring guidelines to local practices.

In Belgium, palliative care in the community is organised in 25 palliative care networks (PCN), set up for populations ranging from 75,000 to 1,200,000 covering the whole territory of Belgium. Primary palliative care at home is led by the patient’s GP and local home care nurses. These primary care teams, and informal caregivers can always call for support of the local PCN. This support consists of advice by phone or of home visits performed by nurses, psychologists and GPs specialised in palliative care.

However, the development of PCN does not guarantee high quality care for all palliative care patients [1]. Therefore, it is imperative to develop primary palliative care. Academic efforts should enhance the knowledge and experience of primary care teams to deliver high quality care to their palliative care patients [2].

Ideally, palliative care starts with early identification of people with potentially incurable disease conditions [3]. However, often palliative care is offered in a late stage of illness, close to death. That is why it is important to study how to implement palliative care as soon as a patient has palliative care needs, regardless of life-expectancy. Internationally, some projects pioneered early identification of palliative care needs and subsequent integrated palliative care [4]. The Belgian ‘Care Pathway for Primary Palliative Care’ (CPPPC) is one of such [4,5]. This CPPPC [5] was developed by the Primary and Interdisciplinary Care Department at the University of Antwerp, inspired by the Gold Standard Framework [6]. The components of the CPPPC are summarised in Table 1 [5,7–9].

**Table 1.** Components of the CPPPC [5].

1) Early identification of palliative care patients with the surprise question [7] and/or the Supportive and Palliative Care Indicator Tool [8]
2) Early assessment using the Palliative Performance Scale [9] anticipatory care planning and a holistic assessment including biological psychological social and existential aspects
3) Interdisciplinary discussion
4) Registration in palliative care pathway file accessible to all team members
5) Follow-up by the team recognising the early, transitional and dying stages within the palliative care continuum.

From 2014 to 2016, the CPPPC was disseminated for implementation in five Belgian areas with financial support of the Belgian National Institute for Health and Disability Insurance. Those areas were two Dutch-speaking PCNs (Antwerp and Limburg), two French-speaking PCNs (Namur and Mons) and also the bilingual region of Brussels [5]. To understand health care implementation issues it is important to know contextual factors [10]. Belgian health care services are mostly fee-for-service, with free access to all levels of care. Though evidence-based guidelines are widely available, there is no quality assurance system for GPs in Belgium. That is why employing guidelines in daily care is not common [11].

This qualitative study was conducted before the attempt to implement the CPPPC. We aimed to assess how palliative care was provided by GPs across Belgium and how the CPPPC could be fitted to the GPs’ needs.

## Method

### Researchers

The University of Antwerp led this project. Among the Antwerp team were three native Dutch-speaking medical doctors, with BVDE having extensive experience, and BL and JW having moderate experience with qualitative research. Two research facilitators of the Antwerp team observed during data collection: SP, a Dutch-speaking psychologist and LF, a bilingual nurse. Native French-speaking qualitative researchers of the Université Catholique de Louvain (a sociologist OS, a public health specialist IA and a nurse MK) conducted and analysed the French-language focus groups. The combination of different perspectives (linguacultural and professional) was expected to be fruitful.

### Design

A multiple-case study including focus groups with GPs of all five regions.

### Sampling and recruitment

The implementation of the CPPPC followed a stepped-wedge cluster design [5,12], starting in another area every six months. This time framework allowed to spread the focus groups over time, after which the CPPPC was implemented by local GPs. Recruitment was done by a trickle-down strategy. First, the PCN and/or the research team called the chairmen of all GP groups in the PCN area asking who of their members would be interested for participation in a focus group to discuss usual palliative care. The research group did not give information about the CPPPC at this stage, to avoid any bias concerning the prior perceptions of the participating GPs about palliative care.

Interested GPs were invited to participate in focus groups. We aimed for diversity of gender, age, experience with palliative care, type of practice, and payment system.

### Data collection

Because these interviews evaluated whether the CPPPC would answer the GPs’ needs, the topic guide (supplemental Appendix 1) was inspired by the CPPPC’s components (Table 1). Open-ended questions explored these palliative care aspects, without pointing directly at ‘quality measures’ used in the analysis.

Each focus group was led and observed by two native speakers. The focus groups were held in quiet meeting rooms and lasted about two hours. Four Dutch-speaking focus groups were held: two in Antwerp (December 2013), one in Brussels (December 2014) and one in Limburg (April 2015). Three French-speaking focus groups were held: one in Mons (April 2014), one in Brussels (May 2015) and one in Namur (September 2015). The focus groups were recorded on tape. Researchers discussed the results after each focus group, and adapted the protocol iteratively based on the findings as discussed in the section ‘Analysis’.

### Analysis

The analysis was informed by two theoretical frameworks related to the quality of palliative care, i.e. the 7Cs of the Gold Standard Framework [6] and six dimensions of quality improvement in health care [10]. The main researcher (BL) set up a first code book in N-Vivo, coding fragments of the first two focus groups conducted in Dutch (Antwerp) into these 13 themes (see Table 2) [6,10]. Sometimes, a participant mentioned a theme that did not seem to belong to one of the predefined themes. Then, a new code was made. The second author (OS) analysed the focus group in French using and adapting the first code book – also in N-Vivo. In a first meeting, the codes were discussed and clarified (BL and OS). Afterwards, BL made a first summary of these first three focus groups, to be discussed by all team members. For the next focus groups, an iterative process of data collection, coding, interpreting, and adapting the protocol was carried out.

**Table 2.** Leading theoretical frameworks for the design of the protocol and the analysis.

Quality of palliative care [6]	Improvement of health care [10]
(C1) Communication	(1) the intervention itself
(C2) Coordination	(2) the professionals working with it
(C3) Control of Symptoms	(3) the patients confronted with it
(C4) Continuity of Care	(4) the social context in which the intervention takes place
(C5) Continued Learning	(5) the administrative/economic/organisational context
(C6) Caregiver Support	(6) the implementation method
(C7) Care in the Dying Phase	

In this iterative process, the protocol was sometimes adapted, especially to ask more questions about some *a priori* themes not spontaneously discussed by the participants (e.g. the dying phase). This stems from a particular perspective on data saturation, aiming for a high degree of *a priori* defined themes being exemplified in the data (‘*a priori* thematic saturation’) rather than achieving data expressing redundantly what previous data *a priori* already had expressed [13].

### Ethical considerations

The study protocol was approved by the Ethical Review Board of Antwerp University Hospital and University of Antwerp (reference 13/35/333). Participants gave their written informed consent before each focus group.

## Results

### Participants

The characteristics of the participants are described in Table 3. Of the 41 participants, 18 cared for fewer than four palliative care patients the year before the focus group (and thus have obviously little exposure to palliative care patients), and 14 participants followed fewer than two educational sessions on palliative care during the previous five years. Nineteen of the 41 participants were older than 55 years and had probably received no training in palliative care while studying medicine.

**Table 3.** Characteristics of the participating family physicians (*n* = 41).

Cluster	Antwerp 1: 3	Antwerp 2: 4	Mons: 7	Brussels-D^1^: 10	Brussels-F^2^: 5	Limburg: 9	Namur: 3
Age category	<36: 4	36–45: 3	46–55: 15	56–65: 13	>65: 6		
Sex	M: 25				F: 16		
Practice category	Single: 11	Duo: 7	Group: 12	Multi-disciplinary: 11			
Payment category	Fee: 30			Capitation: 11			
Number of PC patients last year	0: 2	1–3: 16	4–10: 18	>10: 5			
Times PC education last 5 years	None: 3		Once: 11		More than once: 27		
Location	Urban: 21		Semi-rural: 16		Rural: 4		

^1^Brussels-D: Dutch-speaking GPs in Brussels.

^2^Brussels-F: French-speaking GPs in Brussels.

### Synthesis of themes

Of the 13 predefined themes, the seven themes relating to the four with the GSF [6] were frequently discussed, particularly those related with identification of palliative care needs and breaking bad news. The six themes related to quality improvement [10] were discussed less often, except the impact of patient characteristics and of professional context (particularly everything about collaboration). A predefined theme changed to describe more content was C7 ‘Care in the Dying Phase’. This was changed to C7 ‘Phase-dependent care’ and was divided into five subthemes: ‘Early palliative care phase’, ‘Transitional palliative care phase’, ‘Late/terminal palliative care phase’, ‘Dying phase’, and ‘Mourning phase’. In conclusion, a synthesis of the most compelling findings has been made by both BL and OS. In this synthesis, these seven themes emerged as the most important: first, on the definition of palliative care for GPs (‘what is palliative care for you?’), secondly (very much linked with this definition) whether palliative care can benefit from guidelines and care pathways; then four themes grouped per phase of the palliative care continuum (identification, start-up, handling the taboo and breaking bad news, follow-up), and finally the ever present theme of collaboration.

The most noteworthy are summarised below, with illustrative quotes.

What is palliative care for GPs? Uniqueness of every case – no need for strict guidelines.

GPs considered palliative care to be a process in which every situation is unique. Some feared that guidelines undermine the necessary creativity of health care professionals.

The first experience with advance care planning in the nursing home was this: the nursing staff did ‘tick, tick, tick, tick, tick’ and the computer doesn’t want to close down, because there is one box left to tick (Antwerp 1)

It’s a little bit case by case, there are no rules (Mons)

More than a need for (strict) guidelines, a need for peer review was formulated, and for friendly indications in reflective frameworks.

I think that the most interesting things are not the ex-cathedra things, it is better working on a clinical case in a group of four to five colleagues … because in the end, there is not a lot of choice for end of life drugs, and because no palliative case is like another one. (Brussels- F)

It is interesting to stay cognisant of a few things, indeed by reading guidelines. To make it clear: thought frameworks, reflective frameworks, I want them. (Antwerp 1)

#### Three steps in palliative care:

##### 1. Identification of palliative care patients

Several doctors found it important to identify palliative care patients in a ‘timely’ manner, but admitted that they have difficulties to apply this in practice.

I think that we have always had those conversations, but indeed very late and I sometimes think ‘maybe we could have done that better earlier’. (Antwerp 2)

A limited number of Dutch-speaking GPs used the surprise question (i.e. ’Would you be surprised if this patient died in the next 12 months?’) [7] for screening systematically the palliative care status of their patients – this was not reported by French-speaking GPs. One GP using the surprise question admitted that the inclusion of a patient in the ‘palliative care list’ depends of the individual doctor.

That is doctor by doctor, that is variable. You put that patient on the list, but everyone uses their own criteria for that. (Antwerp 1)

Sometimes, making note of the palliative care phase is done by hospital doctors, who then often transfer the follow-up to the family practitioner very suddenly by occasion of back-referral at home.

Particularly for non-oncological patients it seems difficult to determine when a patient might benefit from a palliative care approach.

##### 2. Starting up palliative care

Even for doctors educated and experienced in palliative care, it is difficult to inform a patient that he is incurably sick. What seems to make it easier is the type of pathology for which palliative care seems to be more evident (cancer), or a situation in which the patients feels that his or her condition is becoming worse.

If the patient himself feels that clearly something is wrong, then they expect from us that we give them a little push in their back and tell them. And if so, then they feel more secure when you raise the matter further and you are able to prepare them. (Limburg)

GPs of both language communities talked about their experiences with advance care planning (ACP), but Dutch-speaking GPs had a more diverse discourse than French-speaking GPs. Dutch-speaking GPs talked more about concrete implementation of early identification and ACP than French-speaking GPs. French-speaking GPs talked about advantages and disadvantages of early identification and ACP, but mostly at a theoretical level.

During meetings of the focus group in Limburg, doctors stated that they would rather use a ‘Care Pathway Complex Care’ than a ‘Care Pathway Palliative Care’, to avoid the palliative label and stigma, and proceed to focus more on care needs of patients than on their life expectancy.

For me, a palliative care patient is a patient who needs complex care. A cardiac failure patient with a need for a lot of care at home, first to evaluate the medical matters, often stopping a lot of medication, for instance statins, and telling the cardiologist that he/she is still having them. Secondly, the non-pharmacological treatment: the layout of the room. Thirdly: who is coming here in the house and is that enough? Should we meet to discuss things? (Limburg)

Finally, and much needed when starting palliative care, ‘handling the taboo’In both language communities, there were doctors for and against the use of the words ‘palliative care’ with patients. In brief, the advocates in favour wanted to reduce the taboo around palliative care by naming it properly, while the others wanted to avoid this terminology to focus on the care itself. Sometimes, doctors discussed very vividly whether or not to use the word ‘palliative care’.

Many doctors in these focus groups expressed a resistance against the formalisation of the palliative care status of a patient. This formalisation would provoke fear in many patients.

My experience is most of all in the nursing home, and the fact is that the word “palliative care” is frightening patients, and even often, the non-educated treating physician. (Mons)

Among French-speaking GPs, this resistance seemed to be a very general one, for any patient, while among Dutch-speaking GPs, resistance to use the word ‘palliative care’ seemed to be stronger for non-cancer patients.

Today there is a strong propensity to have more non-cancer patients in palliative care than cancer patients. Kidney failure, COPD patients, cardiac patients – for me, they are in a palliative phase when there are no invasive treatments possible anymore for good outcomes, that there is in fact a progressive deterioration and that that is complex care, that many people must give support and a lot of medication and various procedures and so on. Actually, I don’t use the word ‘palliative’ there, unless we have a [palliative care] lump sum for it and then I say ‘Look, this is a very unsecure balance and it is going downhill; we will support as much as we can and people are needed for that, so the nurse will come a bit more frequently and so on’. (Limburg)

A good conclusion on this important theme was given by this French-speaking GP:

All the words are good to use, all the truths are good to tell, but there are those words and that moment to tell… … (Namur)

##### 3. Following up palliative care patients at home, always in collaboration

Follow-up of patients in palliative care situations seems very compromised when family members and/or friends do not give the necessary support and informal care.

When do we send people to the hospital in their last days? When that system breaks down; sometimes that happens with singles who have been surrounded very well by friends and neighbours as long as they can still prepare their own bread etc. But there will be a situation where that is not possible anymore, that he is staying in his bed and has to be helped to go to the toilet, that he will fall out of bed at night. (Brussels – D)

Many doctors in these focus groups emphasised that a doctor alone can never deliver optimal palliative care. A lot of attention is given to ‘consensus building with the diverse team players around a patient’. Doctors prefer collaborating with self-designed teams, with trusted home care nurses.

Generally, I think that many doctors have a preferred network of people with whom they work – palliative care nurses, home care nurses in primary care, sometimes also in secondary care. Insofar this is an open choice I think this will go well … if you have everything in your own hands then it goes smoothly, … then everyone knows his place, his role in the story. (Brussels – D)

GPs find they should guarantee a higher level of continuity of care to their palliative care patients than to other patients. The continuity of care out of hours seems to depend on local agreements within a GP group, for instance whether GPs offer out-of-hours medical attention in a classical rota system, or in a general practice cooperative. Many GPs give their personal GSM number to their palliative care patients, and use a home care file to communicate with nurses, also to be used by a doctor on duty. Sometimes the respondents doubted the palliative care competences of their colleagues on duty.

In all focus groups the respondents stated that the transmural collaboration for patients in palliative care situations should be better. Respondents referred to a Belgian study showing organisational problems with oncology team meetings [14]. GPs find it important that a hospital takes into account what the preferences of a GP are for home care nursing – this is important when starting palliative care at home.

## Discussion

### Main findings

GPs expressed a desire for therapeutic freedom. In all seven focus groups, family practitioners were worried that imposing guidelines and standardisation of palliative care according to theoretical ideals would reduce the creativity of ‘tailor-made care’. More than in palliative care protocols, GPs are interested in feedback and peer review methods to improve their own palliative care skills. The phrase ‘palliative care’ is still used with difficulty in all five regions. Some GPs never use these words while others have a personal mission to diminish the palliative care taboo. A third group uses the words ‘palliative care’ if they feel the patient and the family can handle hearing them.

For many GPs, life expectancy is not the main concern when they are starting palliative care, whether it is called palliative care or comfort care. GPs are worried about all their patients in complex care, living with a frail health balance and needing to face the biopsychosocial and spiritual needs of palliative care patients. GPs often struggle with collaboration issues: with other GPs, with paramedical professionals, with hospital-based professionals and so on. The main problem is the difficulty to guarantee continuity of care.

### Training needs exist, but how to respond?

In keeping with our study, a Scottish study evaluating the implementation of a primary palliative care intervention showed the need for training in identification of palliative care patients (especially non-cancer), the need for communication training and the need for enhancement of interdisciplinary communication [15]. GPs in our study expressed a need to provide tailor-made care, which resonates with the reactions on the ‘Liverpool Care Pathway’ leading to the critical review with this eloquent title: More care, less pathway [16]. The lack of interest in ‘strict guidelines’ resonates in other research finding that many clinicians, in diverse settings do not follow guidelines [17]. Feedback and peer review belong to the best ways for GPs to learn palliative care skills [18]. Continuing medical education in palliative care is more and more set up as interdisciplinary and/or with mentoring activities by peers [19,20].

### Palliative care and personal continuity of care

The worries of GPs about all patients with complex needs, not only those at the end of life, is reflected in recommendations stressing the importance of the integration of palliative care within the continuum of advanced chronic conditions [4,21]. An English interview study with 59 bereaved family members about what made death at home possible revealed the importance of continuity of care, particularly personal continuity of care (‘less carers is more’) [22].

In contrast with the importance family care givers give to personal continuity of care, Belgian GPs are changing the organisation of out-of-hours care towards central locations of out-of-hours medical care [23]. These locations offer more possibilities than the classical rota system for a higher quality of palliative care out-of-hours, due to more support in terms of manpower and information logistics – these possibilities are yet to be explored as already happened for instance in the Netherlands [24] and Scotland [25].

### Differences between the regions

Although differences between regions were not investigated from the start, some important differences between Dutch- and French-speaking GPs were identified. The Dutch-speaking GPs talked much about pragmatic problems faced while performing ACP, while French-speaking GPs talked mostly about theoretical problems with ACP. This finding confirms that palliative care practices differ per language community, as previously suggested [21,26]. A plausible explanation for these differences is that Dutch-speaking and French-speaking communities are only loosely connected. Even though Belgium has a federal health insurance, the policies for health care provider education and primary care implementation are regionalised [27]. Dutch-speaking and French-speaking GPs never meet each other in educational sessions or other professional meetings, except in Brussels.

For the promotion of ACP, the priority in the French-speaking provinces is promoting general communication techniques of ACP, while in Dutch-speaking provinces educative initiatives for specific target groups can be organised as well as general initiatives.

### Strengths and limitations

Conducting focus groups seems the best way of exploring perceptions of ‘usual care’, because they are able to reveal subtle differences between individuals of ideas about a theme and can give insights in the diversity of practice. The researchers formed a multidisciplinary team with experience in qualitative research. Although there were two language groups to be investigated, all focus groups were moderated and observed by native speakers.

These focus groups generated rich data. Almost half of the participants had fewer than four palliative care cases the year before the focus group and had followed fewer than two palliative care classes the last five years. This is both a limitation (not much experience or interest in palliative care post-graduate education) and a strength (these participants tell how ‘common’ GPs without a special interest in palliative care think about this part of their job). The focus groups were sometimes very small (twice only 3 GPs), this possibly limited their data. Elsewhere we published reasons for non-participation for the implementation part of this project [28].

Though this article is published 4–5 years after the focus groups, the results are still relevant. Key stakeholders in primary palliative care did not change, there were no effective changes in the law on palliative care, and there were no large-scale campaigns on palliative care.

### Implications: how to present palliative care guidelines to GPs?

To meet the need of GPs in delivering continuous ‘tailor-made’ care, palliative care guidelines must recommend a personalised and patient-centred approach. Evidence-based medicine is the integration of best research evidence with clinical expertise and patient values [29].

This philosophy was followed in how the CPPPC was presented, stressing that patient experience is one of four types of evidence informing evidence-based care [30]. By using the CPPPC identification and follow-up tools, it should be possible to scale-up personally tailored care with attention for continuity of care in all its aspects, supported by a whole team [31].

Any guideline on itself does not meet the needs of GPs concerning their communications skills, palliative care knowledge and attitudes, and professional networking, but during the CPPPC lectures the GPs were stimulated in these elements as well.

### Suggestion for future research

A follow-up study could be performed in several European countries, including different cultures and health systems. This approach would make it possible to verify whether some of the conclusions of this study are relevant throughout Europe.

## Conclusion

Belgian GPs want to deliver ‘tailor-made’ palliative care, in freedom. They lack the expertise to translate evidence-based guidance in patient-oriented care and fail to meet patients’ preferences by proven best clinical practices. Rather than disseminating guidelines like the CPPPC, suggested strategies to improve the role of GPs in palliative care are peer reviews of palliative care cases, and practice-oriented education which can offer reflective frameworks around ‘what is adequate palliative care?’

## Supplementary Material

Supplemental Material: Appendix 1 - Topic Guide
