# Supplementary material for: Implementation of Primary Palliative Care in five Belgian regions: A qualitative study on early identification of palliative care needs by general practitioners
Source: Eur J Gen Pract. 2020 Oct 20;26(1):146–53. doi: 10.1080/13814788.2020.1825675 (PMC7592891; doi:10.1080/13814788.2020.1825675)
Supplement: Supplemental Material: Appendix 1 - Topic Guide [file IGEN_A_1825675_SM4618.docx]

**Appendix 1. Topic guide**

1. Opening phrase: Thank you for being here with us in this focus group. We know it is easy to give time to research activities when you have a busy clinical practice and we appreciate you coming here. The region where you work has accepted to implement the CPPPC.

We would like to investigate whether the CPPPC can be a good tool to improve the quality of palliative care. To be able to do that, we need GPs to tell us how GPs perform palliative care without knowing the CPPPC.

Today we want to focus with this group on how you as GPs experience providing care to your palliative patients and which problems arise when doing that. Your own professional experiences are very valuable for us. There are no bad answers.

Information about audio taping and informed consent.

1. Warming up: GPs present themselves to each other + fill paper with background data
2. **First item**: is early recognition of palliative care patients and early breaking of bad news (“you are a palliative patient”) important for GPs?

- Chief question: How do you start a palliative care phase? Is the change to the palliative care communicated with the patient? When and how do you tell patients that palliative care should be given…
- Added question 1: Is it important for you to begin this type of conversations early? With ‘early’ we mean: when the life-expectancy is more or less a year (cf. “surprise question”: would I be surprised if a patient would die within this year?)
- Added question 2: Is it important for you to begin this type of conversations for every patient, no matter which categories of diseases he or she has?
- Added question 3: Is identification of palliative patients done intuitively in the direct contact with patients or do you sometimes use a more systematic approach?
- Added question 4: Is the word ‘palliative’ easily used during advance care planning conversations? How do you tell you some in an anticipatory way that his of her life has come on the ‘volta’ of curative and palliative care?

1. **Second item**: competences needed to start palliative care

- Chief question: In the ‘ideal world’ we can break the bad news of ‘you are a palliative patient we will do everything to comfort you’ to all patients with a reduced life expectancy. Does it go like that in real life?
- Added question 1: When does this go well? And why?
- Added question 2: When is it a hard thing to do? And why?
- Added question 3: Do you feel well prepared to start palliative care?
- Added question 4: Would a guideline or another decision supporting instrument to start palliative care be welcome?

1. **Third item**: competences needed for follow-up of palliative patients
   1. Chief question: How do you follow palliative patients now? (how often, when, who takes the initiative, …)
   2. Added question 1: When changes the frequency of follow-up?
   3. Added question 2: What aspects of care do you or your team provide to patients (somatic/psychological/social/existential)? What goes well? And what doesn’t go so well?
   4. Added question 3: What moment is OK for you to ask for the palliative forfeit? How do you recognize this moment?
   5. Added question 4: Would a guideline or another decision supporting instrument to follow palliative patients be welcome?
2. **Fourth item**: competences needed for collaborating with other primary health care workers
   1. Chief question: Palliative care is complex, and can often not be provided in all its dimensions by only one health care professional. How do you, as GPs, organize the collaboration with other health care workers? How is the family involved in this “team”?
   2. Added question 1: If there is a care discussion about a patient’s case, is this done in a regular, organized way or is it rather organized ad hoc? And how do you feel about that?
   3. Added question 2: How do these care discussions become concrete? Via a shared care file, contact via email and/or telephone, or in an organized care discussion? And how do you feel about that?
   4. Added question 3: If you as a GP notice that the current care for a specific patient is “not enough”, how do you solve this? With or without discussing this with others?
   5. Added question 4: Do you know the differing possibilities to support palliative patients and do you suggest these options to your patients? E.g. palliative care network’s accessibility by phone, support by volunteers of the palliative care network, palliative residential unit.
